# Clinical characteristics of Mycoplasma pneumoniae pneumonia in children with atopic constitution and risk factors for disease severity: a retrospective comparative study

**DOI:** 10.3389/fped.2026.1760932

**Published:** 2026-04-10

**Authors:** Yuehu Liu, Yuan Fang, Peng Chen, Wenqing Li, Xiaoxia Lu

**Affiliations:** 1 Department of Respiratory Medicine, Wuhan Children's Hospital, Tongji Medical College, Huazhong University of Science and Technology, Wuhan, China; 2 Department of General Internal Medicine II, Wuhan Children's Hospital, Tongji Medical College, Huazhong University of Science and Technology, Wuhan, China; 3 School of Nursing, Wuhan University, Wuhan, China

**Keywords:** atopic constitution, children, clinical characteristics, Mycoplasma pneumonia, severe pneumonia

## Abstract

**Objective:**

To compare the clinical characteristics and immune-inflammatory markers between Mycoplasma pneumoniae pneumonia children with and without atopic constitution, and to identify independent risk factors for severe MPP within the atopic group.

**Methods:**

We retrospectively analyzed the medical records of 377 children diagnosed with MPP at Wuhan Children's Hospital between January 1, 2019, and December 31, 2024, who underwent allergen-specific immunoglobulin E (sIgE) testing for both inhalant and food allergens. The children were divided into two groups based on their atopic constitution: the atopic constitution group (*n* = 282) and the non-atopic constitution group (*n* = 95). General data, clinical symptoms, and laboratory test results were compared between the two groups. Logistic regression was used to identify independent risk factors for severe MPP in children with an atopic constitution, and ROC curves were used to determine the cutoff values for predictive markers.

**Results:**

Among the 377 children with MPP, 282 (74.8%) were identified as having an atopic constitution. Within the atopic constitution group (*n* = 282), 20 children (7.1%) progressed to severe Mycoplasma pneumoniae pneumonia (SMPP). No significant differences were observed between the atopic and non-atopic groups regarding age, fever duration, or length of hospital stay (*P* > 0.05). However, the atopic group showed a significantly higher incidence of wheezing (48.6% vs. 25.3%), stridor (34.4% vs. 20.0%), and dyspnea (15.2% vs. 4.2%) (*P* < 0.05). Multivariable logistic regression analysis revealed that oxygen therapy (OR = 57.49, 95% CI: 1.69–1,952.29, *P* = 0.024), a history of corticosteroid use (OR = 9.28, 95% CI: 1.19–72.15, *P* = 0.033), serum ferritin > 107.61 ng/mL (OR = 1.01, 95% CI: 1.01–1.02, *P* < 0.05), and IgE > 1,060 IU/mL (OR = 3.16, 95% CI: 1.10–9.07, *P* = 0.032) were independent risk factors for severe MPP in children with atopic constitution. The ROC curve indicated that serum ferritin had the highest predictive performance (AUC = 0.75, 95% CI: 0.60–0.89).

**Conclusion:**

Children with an atopic constitution exhibited a significantly higher prevalence of airway hyperreactivity symptoms during Mycoplasma pneumoniae infection compared to those without an atopic background. Clinically, high serum ferritin and IgE levels should be considered important independent risk factors for predicting severe MPP in children with an atopic constitution, which could facilitate early intervention and improve prognosis.

## 1 Introduction

According to the World Allergy Organization, as the prevalence of allergic diseases increases, approximately 30%–40% of the global population is affected, with a higher incidence in children from industrialized countries. Children with atopic constitution often suffer from one or more of the following diseases: atopic dermatitis, allergic asthma, food allergies, or allergic rhinitis (1, 2). Mycoplasma pneumoniae pneumonia (MPP) accounts for 10%–40% of community-acquired pneumonia in children. Some infected children may develop extrapulmonary complications and may be left with sequelae such as atelectasis, bronchiectasis, obstructive bronchitis, and obstructive bronchiolitis, which can affect the child's long-term health and quality of life (3). Respiratory pathogen infections are closely related to and are intertwined with allergic diseases in children. Mycoplasma infection is an important factor in asthma exacerbation. The mechanisms by which it worsens and triggers asthma attacks mainly include airway epithelial damage, activation of innate immunity, and an enhanced Th2-dominated immune response (4). This study evaluates the impact of atopic constitution on the clinical and immunological progression of MPP. By comparing atopic and non-atopic cohorts and delineating severity predictors within the atopic group, we aim to refine early identification and targeted intervention strategies for high-risk pediatric patients.

## 2 Materials and methods

### 2.1 Study subjects

This retrospective study included 377 hospitalized children diagnosed with MPP at Wuhan Children's Hospital between January 1, 2019, and December 31, 2024. The study participants were primarily infants and young children. All participants underwent inhalant and food allergen sIgE testing. Based on the presence or absence of an atopic constitution, the children were divided into two groups: the atopic and non-atopic constitution groups. This study was approved by the Ethics Committee of Wuhan Children's Hospital (Approval Number: 2025R086-E01).

### 2.2 Inclusion criteria

According to the national guidelines for the diagnosis and treatment of MPP, the inclusion criteria were as follows: (1) a single serum Mycoplasma pneumoniae (MP) antibody titer ≥1:160 (determined by the Particle Agglutination assay) or a fourfold increase in the MP antibody titer between two serum samples during the course of illness. (2) Positive Mycoplasma pneumoniae deoxyribonucleic acid (DNA) or ribonucleic acid (RNA) detected in nasopharyngeal swabs or bronchoalveolar lavage fluid (BALF) samples.

### 2.3 The exclusion criteria

Were as follows: (1) incomplete clinical data; (2) IgE-type multiple myeloma, bronchopulmonary mycosis, parasitic infections, or other diseases causing IgE elevation; (3) children who have undergone specific immunotherapy; and (4) children with chronic diseases such as congenital metabolic disorders or immune deficiencies.

### 2.4 Allergen testing

The allergen-specific IgE (sIgE) levels were measured using a comprehensive panel of 20 allergens.

#### 2.4.1 Food allergen panel (10 items)

Peanut (Arachis hypogaea), Soybean (Glycine max), Cow's milk, Crab, Shrimp, Egg, Beef, Cod fish, Wheat flour, and Tomatoes.

#### 2.4.2 Inhalant allergen panel (10 items)

Short Ragweed (Ambrosia artemisiifolia), Mugwort (Artemisia vulgaris), House Dust Mite (Dermatophagoides pteronyssinus), Dust Mite (Dermatophagoides farinae), Cat dander, Dog dander, House Dust, Cockroach, Alternaria alternata, and Willow (Salix).

*Note: In this study, “House Dust Mites’ refers to the specific biological species (D. pteronyssinus and D. farinae), whereas ‘House Dust’ refers to the general indoor environmental dust mixture.*”.

### 2.5 Atopic constitution diagnostic criteria

In this study, children were classified as having an atopic constitution if they satisfied **at least one** of the following criteria (separated by the logical “**OR**”): (1) A history of recurrent eczema or wheezing, or a history of allergic diseases such as allergic asthma, allergic rhinitis, or atopic dermatitis; (2) at least one positive result for allergen-specific IgE in the past six months or a total IgE level greater than 200 IU/mL; (3) positive results from food provocation tests or skin prick tests (5).

### 2.6 Diagnosis of severe pneumonia

The severity of pneumonia in all children included in this study was assessed according to the National Community-Acquired Pneumonia Guidelines for Children (2019 edition) (6).

### 2.7 Diagnosis of severe Mycoplasma pneumonia

According to the 2019 edition of the “Diagnosis and Treatment Specifications for Community-Acquired Pneumonia in Children,” and based on a confirmed diagnosis of MPP, the 2023 edition of the “Diagnosis and Treatment Guidelines for Mycoplasma Pneumonia in Children” (7) establishes the following criteria for severe mycoplasma pneumonia: any one of the following clinical manifestations must be present: 1. Persistent high fever (≥39 °C) for ≥5 days or fever for ≥7 days with no downward trend in peak temperature; 2. Presence of one of the following: wheezing, shortness of breath, dyspnea, chest pain, or hemoptysis. These manifestations are associated with severe lesions, coexisting plastic bronchitis, asthma attacks, pleural effusion, or pulmonary embolism; 3. Development of extrapulmonary complications that do not meet critical illness criteria; 4. Oxygen saturation ≤0.93% at rest while breathing room air. 5. Imaging findings meeting any of the following: (1) Involvement of ≥2/3 of a single lobe with uniform high-density consolidation, or high-density consolidation in two or more lobes (regardless of extent), possibly accompanied by moderate to massive pleural effusion and localized bronchiolitis; (2) Diffuse bronchitis in ≥4/5 lobes of one lung or both lungs, possibly with bronchitis, accompanied by atelectasis due to mucus plug formation. 6. Progressive worsening of clinical symptoms with imaging showing >50% increase in lesion extent within 24–48 h. 7. Significant elevation in any one of CRP, LDH, or D-dimer.

### 2.8 Data collection

Data for this study were retrieved from the hospital's electronic medical record system. A standardized form was designed to record the clinical features of each child. The collected data included basic information such as sex, age, and testing date, as well as the results of inhalant and food allergen sIgE testing, clinical symptoms (e.g., fever, wheezing, nasal congestion, runny nose, moist rales, stridor, dyspnea), signs (e.g., cyanosis, vomiting, diarrhea), underlying diseases (e.g., congenital heart disease, bronchopulmonary dysplasia), and final clinical diagnoses. Laboratory test results (e.g., serum IgE, erythrocyte sedimentation rate, creatine kinase, lactate dehydrogenase, liver and kidney function tests, and cytokine levels), imaging studies (e.g., chest x-rays), and treatment information (e.g., corticosteroid use, oxygen therapy, and length of stay) were also recorded. All data were organized in a standardized table to ensure consistency and completeness.

### 2.9 Statistical analysis

Statistical analyses were performed using SPSS 29.0 software. Normally distributed continuous data were expressed as mean ± standard deviation (Mean ± SD), while non-normally distributed data were expressed as median (interquartile range) [M(Q1, Q3)]. For normally distributed data with homogeneity of variance, comparisons between groups were made using independent *t*-tests; otherwise, the Mann–Whitney *U*-test was used. Categorical data were expressed as frequencies and percentages, and group comparisons were performed using the chi-square test (Categorical variables were compared using the Pearson chi-square test or Fisher's exact test, as appropriate). Univariate and multivariable logistic regression analyses were used to explore the risk factors for severe MPP in children with an atopic constitution. Statistical significance was set at *P* < 0.05. Statistical analyses were performed using the R version 4.3.3.

## 3 Results

### 3.1 Allergen sensitization distribution in children

**Figure 1:**
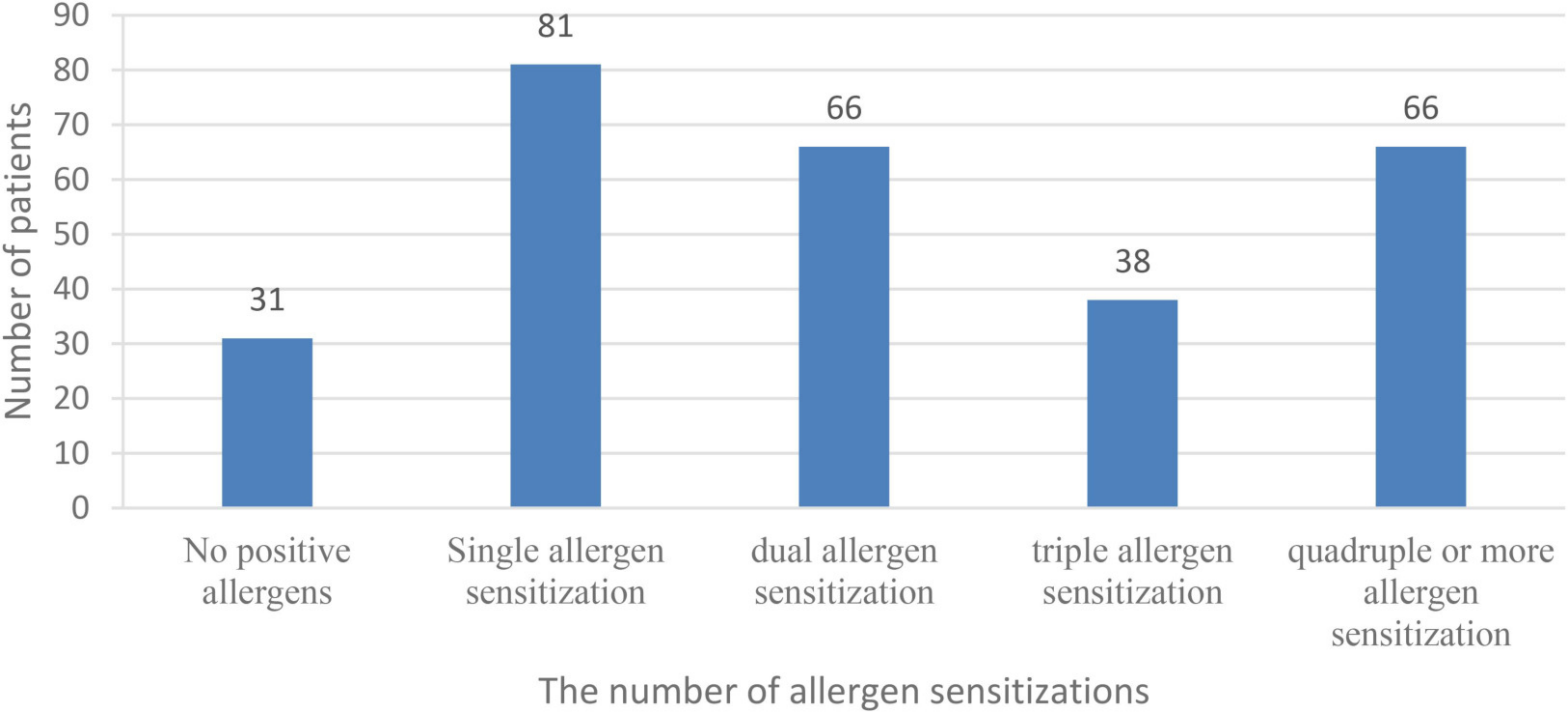
Allergen sensitization Status in children with atopic constitution and Mycoplasma Pneumoniae pneumonia.

**Figure 2:**
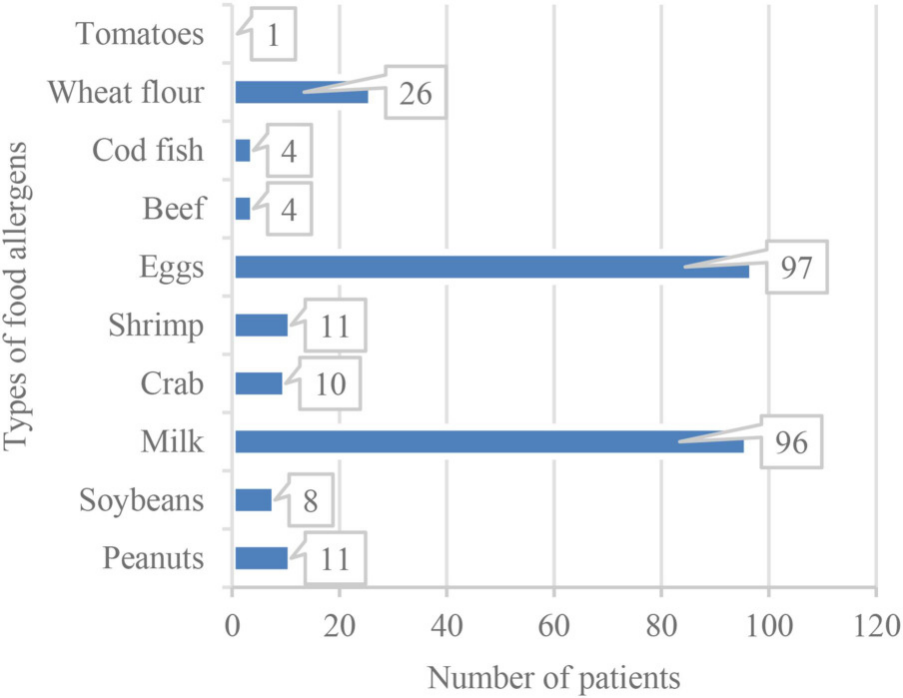
Distribution of positive results for food allergens.

**Figure 3:**
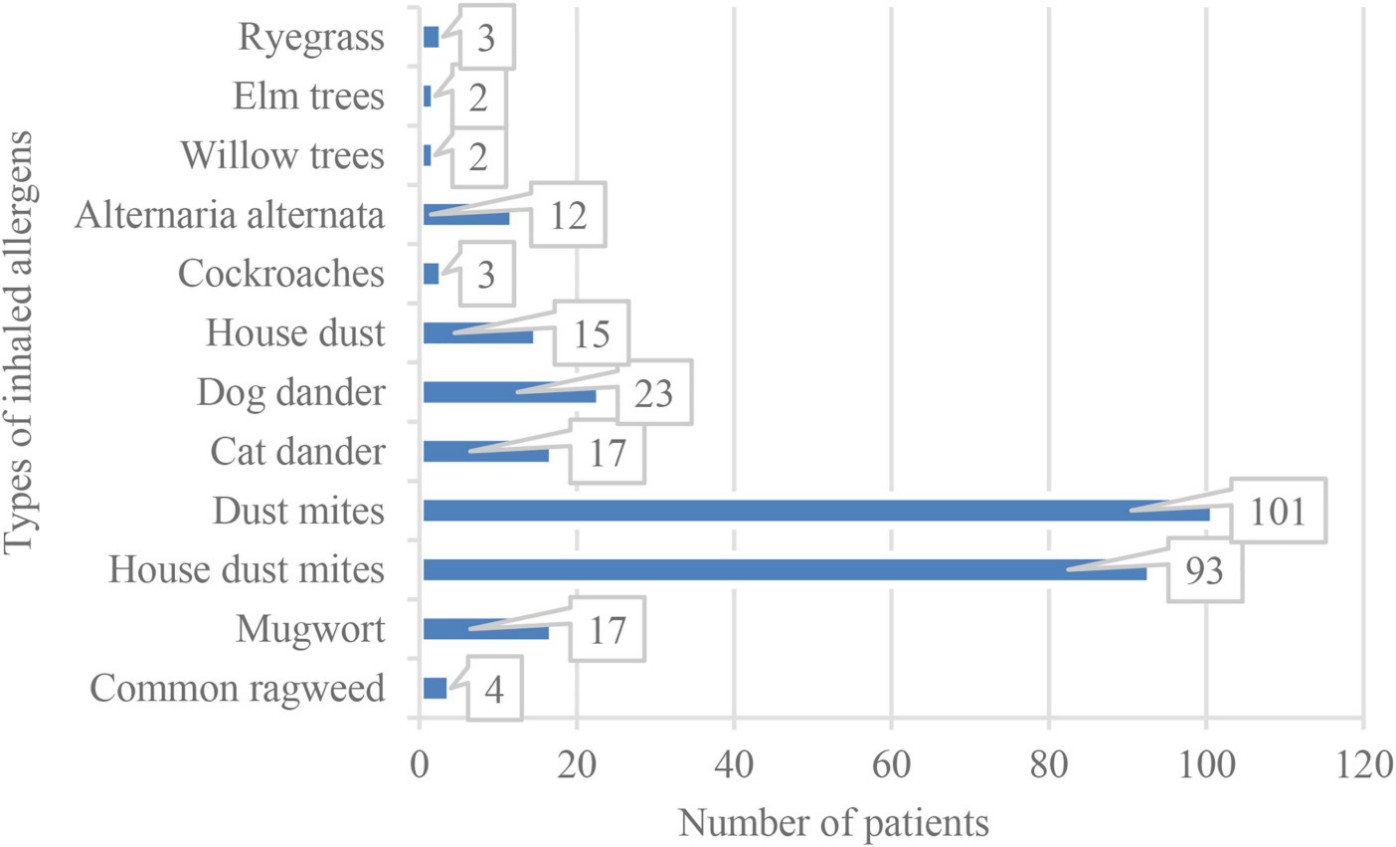
Distribution of positive results for inhalant allergens.

This study included 282 children with atopic constitution and MPP. The distribution of allergen sensitization types is shown in Figure 1. The majority of children were sensitized to a single allergen (81 cases), followed by dual and multiple allergen sensitivities, with 66 cases each for dual sensitization and more than four allergens each. Thirty-eight children had triple allergen sensitivity. The fewest number of children had no positive allergen tests, with only 31 cases (Figures 1–3).

In food allergen testing, 268 positive cases were detected. As shown in Figure 2, the most common positive food allergens were eggs (97 cases, 36.19%), milk (96 cases, 35.82%), and wheat flour (26 cases, 9.70%). Other allergens included shrimp (11 cases), crabs (10 cases), soy (8 cases), and peanuts (11 cases). Beef and cod fish each had four positive cases (1.49%), while tomatoes had the lowest positive detection rate (1 case, 0.37%).

In inhalant allergen testing, 292 positive cases were detected. As shown in Figure 3, children had the highest sensitization rates for dust mite allergens. Among these, dust mites (101 cases, 34.6%) and house dust mites (93 cases, 31.8%) were the most frequently detected. The third most common allergen was dog dander (23 patients, 7.9%). Other inhalant allergens included mugwort (17 cases, 5.8%), cat dander (17 cases, 5.8%), house dust (15 cases, 5.1%), and Alternaria alternata (12 cases, 4.1%). Common ragweed had 4 cases (1.4%), whereas cockroach and ryegrass each had 3 cases (1.0%). The lowest detection rate was for willow and elm trees, with two cases (0.7%) each.

### 3.2 Comparison of general and clinical data

There were no significant differences between the atopic and non-atopic groups in terms of age, fever duration, length of hospital stay, incidence of extrapulmonary complications, lobar pneumonia, or bronchopneumonia (all *P* > 0.05). However, in terms of clinical symptoms and signs, the atopic group showed significantly higher rates of wheezing (48.6% vs. 25.3%, *χ*^2^ = 15.792, *P* < 0.05), stridor (34.4% vs. 20.0%, *χ*^2^ = 6.915, *P* = 0.010), and dyspnea (15.2% vs. 4.2%, *χ*^2^ = 7.933, *P* = 0.006). Although differences in chronic airway disease comorbidities (*P* = 0.052), oxygen therapy (*P* = 0.105), and corticosteroid use (*P* = 0.058) did not reach statistical significance, the proportions of patients in the atopic constitution group were higher than those in the non-atopic constitution group (Table 1).

**Table 1:** Comparison of general and clinical data between atopic and non-atopic constitution groups.

Item	Atopic constitution group (*n* = 282)	Non-atopic constitution group (*n* = 95)	*Z*/*χ*^2^ value	*P* value
Age [M(Q1, Q3), months]	4.83 (3.33, 7.00)	5.03 (3.46, 7.12)	0.426	0.670
Fever Duration [M(Q1, Q3), days]	5.00 (3.00, 7.00)	5.00 (3.00, 7.00)	1.196	0.232
Hospital Stay [M(Q1, Q3), days]	5.00 (4.00, 7.00)	5.00 (4.00, 7.00)	0.530	0.596
Wheezing [*n* (%)]	137 (48.6%)	24 (25.3%)	15.792	<0.001
Nasal Congestion [*n* (%)]	43 (15.2%)	15 (15.8%)	0.016	1.000
Runny Nose [*n* (%)]	51 (18.1%)	17 (17.9%)	0.002	1.000
Moist Rales [*n* (%)]	95 (33.7%)	24 (25.3%)	2.335	0.160
Stridor [*n* (%)]	97 (34.4%)	19 (20.0%)	6.915	0.010
Dyspnea [*n* (%)]	43 (15.2%)	4 (4.2%)	7.933	0.006
Cyanosis [*n* (%)]	1 (0.4%)	0 (0.0%)	0.338	1.000
Vomiting [*n* (%)]	30 (10.6%)	8 (8.4%)	0.385	0.565
Diarrhea [*n* (%)]	3 (1.1%)	3 (3.2%)	1.99	0.171
Extrapulmonary Complications [*n* (%)]	5 (1.8%)	2 (2.1%)	0.043	1.000
Chronic Airway Disease [*n* (%)]	119 (42.2%)	29 (30.5%	4.06	0.052
Lobar Pneumonia [*n* (%)]	143 (50.7%)	47 (49.5%)	0.043	0.906
Bronchopneumonia [*n* (%)]	89 (31.6%)	35 (36.8%)	0.898	0.377
Allergic History [*n* (%)]	251 (89.0%)	0 (0.0%)	252.99	<0.001
Pleural Effusion [*n* (%)]	10 (3.5%)	2 (2.1%)	0.479	0.738
Family Allergic History [*n* (%)]	13 (4.6%)	2 (2.1%)	1.167	0.374
Oxygen Therapy [*n* (%)]	40 (14.2%)	7 (7.4%)	3.025	0.105
Corticosteroid Use [*n* (%)]	54 (19.1%)	10 (10.5%)	3.749	0.058
Bronchoscopy + Bronchoalveolar Lavage [*n* (%)]	17 (6.0%)	8 (8.4%)	0.657	0.474
Severe MPP [*n* (%)]	20(7.1%)	6(6.3%)	0.568	0.504

### 3.3 Comparison of laboratory test results

There were no significant differences between the two groups in most laboratory indicators, including erythrocyte sedimentation rate, myocardial enzyme profile, liver enzymes, kidney function, D-dimer, lymphocyte subgroups, multiple cytokines, vitamin D, serum ferritin, and acute-phase proteins (SAA and CRP) (all *P* > 0.05). However, the total immunoglobulin E (IgE) levels in the atopic constitution group were significantly higher than those in the non-atopic constitution group (*P* < 0.05), whereas their lymphocyte count was significantly lower (*P* < 0.05). Furthermore, procalcitonin (PCT) levels (*P* = 0.010) were significantly lower, whereas tumor necrosis factor-alpha (TNF-α) levels (*P* = 0.048), hemoglobin concentration (*P* = 0.003), and platelet count (*P* = 0.023) were significantly higher in the atopic group (Table 2).

**Table 2:** Comparison of laboratory test results between children with atopic and non-atopic constitution.

Item	Atopic constitution group (*n* = 282)	Non-atopic constitution group (*n* = 95)	Z/*χ*^2^ Value	*P* Value
Erythrocyte Sedimentation Rate [M(Q1, Q3), mm/h]	15.00 (7.0, 28.00)	21.00 (8.50, 36.00)	−1.468	0.142
Creatine Kinase [M(Q1, Q3), U/L]	94.00 (64.00, 132.00)	89 (53.50, 108.00)	−1.4	0.161
CK-MB[M(Q_1_,Q_3_), U/L]	28.00 (21.00, 39.00)	31.00 (22.50, 59.00)	−0.373	0.709
LDH[M(Q_1_,Q_3_), U/L]	304.00 (266.00, 354.00)	300.00 (238.00, 415.00)	−0.805	0.421
Urea Nitrogen [mmol/L]	3.29 (0.92)	3.64 (0.63)	0.172	0.864
Creatinine [M(Q_1_,Q_3_), μmol/L]	30.90 (24.90, 37.40)	29.40 (25.25, 37.00)	−0.954	0.34
ALT[M(Q_1_,Q_3_), U/L]	11.00 (9.00, 16.00)	9.00 (6.50, 17.00)	−0.965	0.335
AST[M(Q_1_,Q_3_), U/L]	29.00 (25.00, 35.00)	25.00 (22.50, 38.50)	−1.508	0.132
Albumin [g/L]	43.00 (41.50, 45.50)	42.50 (41.20, 43.95)	0.687	0.246
D-Dimer [M(Q_1_,Q_3_), mg/L FEU]	0.29 (0.16, 0.43)	0.25 (0.15, 0.82)	−0.061	0.951
CD16 + CD56[M(Q_1_,Q_3_), %]	8.77 (5.66, 13.39)	8.55 (5.66, 13.37)	−0.166	0.868
CD19[M(Q_1_,Q_3_), %]	20.55 (15.40, 27.04)	21.40 (6.38)	−0.032	0.974
CD3[M(Q_1_,Q_3_), %]	66.72 (59.69, 72.54)	66.76 (8.09)	−0.762	0.446
CD3 + CD4[μmol/L, %]	34.46 (8.19)	36.61 (6.56)	−1.582	0.116
CD4[μmol/L, %]	37.12 (6.41)	36.13 (7.45)	0.769	0.221
CD8[M(Q_1_,Q_3_), %]	24.32 (19.56, 27.94)	25.94 (21.93, 28.47)	−0.893	0.372
CD4/CD8[M(Q_1_,Q_3_), %]	1.52 (1.23, 1.87)	1.32 (1.18, 1.73)	−0.14	0.888
Procalcitonin [M(Q_1_,Q_3_), ng/mL]	0.1 (0.08, 0.13)	0.16 (0.12)	−2.561	0.01
IFN-*γ*[M(Q_1_,Q_3_), pg/mL]	5.97 (3.81, 8.03)	9.71 (3.94, 255.67)	−0.431	0.666
IL-10[M(Q_1_,Q_3_), pg/mL]	6.50 (5.28, 9.09)	6.07 (4.28, 18.91)	−1.143	0.253
IL-2[M(Q_1_,Q_3_), pg/mL]	3.27 (2.29, 4.50)	2.21 (1.58, 4.69)	−0.471	0.637
IL-4[M(Q_1_,Q_3_), pg/mL]	3.40 (2.94, 3.94)	3.56 (2.25)	−0.609	0.542
IL-6[M(Q_1_,Q_3_), pg/mL]	23.86 (14.79, 125.16)	21.43 (12.88, 310.17)	−0.711	0.477
TNF-α[M(Q_1_,Q_3_), pg/mL]	3.79 (2.54, 4.36)	2.47 (0.97, 4.55)	−1.978	0.048
25-Hydroxy Vitamin D [M(Q_1_,Q_3_), ng/ml]	32.97 (27.25, 42.23)	30.53 (22.20, 38.35)	−0.823	0.411
Serum Ferritin [M(Q_1_,Q_3_), ng/mL]]	75.86 (65.13, 110.69)	82.39 (60.48, 108.07)	−0.034	0.973
IgE[M(Q_1_,Q_3_), g/L]	229.00 (131.75, 713.00)	48.10 (23.40, 101.00)	−9.821	<0.001
IgA[M(Q_1_,Q_3_), g/L]	1.16 (0.67, 1.88)	1.32 (0.68)	−0.219	0.827
IgG[M(Q_1_,Q_3_), g/L]	8.69 (7.40, 10.88)	9.82 (2.68)	−1.023	0.306
IgM[g/L]	1.40 (0.51)	1.22 (0.98, 1.76)	−1.039	0.299
Leukocyte Count [M(Q_1_,Q_3_), ×10^9^/L]	9.49 (7.36, 11.37)	7.21 (5.46, 9.34)	−0.609	0.542
Neutrophil Count [M(Q_1_,Q_3_), ×10^9^/L]	59.67 (46.50, 69.58)	54.48 (17.04)	−1.491	0.136
Lymphocyte Count [M(Q_1_,Q_3_), ×10^9^/L]	28.35 (22.40, 37.15)	36.25 (16.41)	−3.421	<0.001
Red Blood Cell Count [M(Q_1_,Q_3_), ×10^12^/L]	4.66 (4.40, 4.83)	4.50 (4.23, 4.72)	−2.182	0.564
Hemoglobin Concentration [M(Q_1_,Q_3_), g/L]	127.00 (121.75, 137.75)	124.52 (10.55)	−2.962	0.003
Platelet Count [M(Q_1_,Q_3_), ×10^9^/L]	328.00 (263.25, 367.50)	279.00 (209.00, 332.00)	−2.275	0.023
Serum Amyloid A [M(Q_1_,Q_3_), mg/L]	43.21 (19.31, 120.14)	20.82 (9.05, 105.20)	−0.736	0.462
C-Reactive Protein [M(Q_1_,Q_3_), mg/L]	7.00 (3.31, 16.01)	7.37 (3.00, 13.66)	−0.459	0.646

### 3.4 Chronic airway disease comorbidities in atopic vs. non-atopic constitution groups

Children in the atopic constitution group had a higher comorbidity rate of chronic airway diseases, with a significant number having a history of wheezing (15.12%) and of rhinitis (13.79%). In the atopic constitution group, there were also cases of asthma (six cases), obstructive bronchiolitis (two cases), cough-variant asthma (one case), and primary ciliary dyskinesia (one case). In contrast, the non-atopic constitution group had significantly lower comorbidities, primarily consisting of recurrent respiratory infections (1.86%), wheezing (1.59%), and sinusitis (0.80%), which are not allergy-related (Table 3).

**Table 3:** Comorbidities of chronic airway diseases in atopic and Non-atopic constitution groups.

Chronic airway disease name	Total cases	Atopic constitution group cases (*n* = 282)	Percentage	Non-atopic constitution group cases (*n* = 95)	Percentage
Rhinitis	54	52	13.79%	2	0.53%
Wheezing History	63	57	15.12%	6	1.59%
Recurrent Respiratory Infections	24	17	4.51%	7	1.86%
Primary Ciliary Dyskinesia	1	1	0.27%	0	0
Bronchial Asthma	6	6	1.59%	0	0
Eczema	20	18	4.77%	2	0.53%
Sinusitis	4	1	0.27%	3	0.80%
Chronic Cough	3	1	0.27%	2	0.53%
Pneumonia	5	3	0.80%	2	0.53%
Obstructive Bronchiolitis	2	2	0.53%	0	0
Cough Variant Asthma	1	1	0.27%	0	0

### 3.5 Risk factors for severe MPP in children with atopic constitution

#### 3.5.1 Univariate logistic regression analysis

To further understand why certain children with an atopic constitution develop severe disease, we performed a subgroup analysis of the 282 atopic patients, comparing those with severe MPP (*n* = 20) to those with non-severe MPP (*n* = 262) (Table 4). Univariate logistic regression analysis of risk factors for severe MPP in children with an atopic constitution showed that several clinical and laboratory indicators were significantly associated with the risk of severe MPP (*P* < 0.05). The clinical risk factors included dyspnea (OR = 3.38, 95% CI: 1.26–9.04, *P* = 0.015), extrapulmonary complications (OR = 22.94, 95% CI: 3.59–146.67, *P* < 0.05), lobar pneumonia (OR = 6.12, 95% CI: 1.75–21.37, *P* = 0.005), pleural effusion (OR = 6.43, 95% CI: 1.52–27.10, *P* = 0.011), oxygen therapy (OR = 7.73, 95% CI: 2.97–20.11, *P* < 0.05), history of corticosteroid use (OR = 6.22, 95% CI: 2.43–15.93, *P* < 0.05), bronchoscopy (OR = 6.94, 95% CI: 2.16–22.29, *P* = 0.001), and length of hospital stay (OR = 1.46, 95% CI: 1.17–1.84, *P* = 0.001). Among the laboratory quantitative indicators, serum ferritin (OR = 1.01, 95% CI: 1.01–1.02, *P* < 0.05), IgE (OR = 1.01, 95% CI: 1.01–1.01, *P* = 0.036), and neutrophil counts (OR = 1.03, 95% CI: 1.01–1.07, *P* = 0.037) were positively correlated with the risk of severe MPP. Bronchopneumonia (OR = 0.22, 95% CI: 0.05–0.99, *P* = 0.048) and a history of allergen sensitization (OR = 0.33, 95% CI: 0.11–0.98, *P* = 0.047) were identified as protective factors.

**Table 4:** Univariate logistic regression analysis of risk factors for severe MPP in children with atopic constitution.

Factor	*P* Value	Univariate analysis OR (95%CI)
Wheezing	0.213	0.55 (0.21–1.41)
Nasal Congestion	0.542	1.43 (0.45–4.50)
Runny Nose	0.339	0.48 (0.11–2.15)
Moist Rales	0.718	0.83 (0.31–2.24)
Stridor	0.953	1.03 (0.40–2.67)
Dyspnea	0.015	3.38 (1.26–9.04)
Vomiting	0.410	0.42 (0.05–3.28)
Diarrhea	0.123	6.84 (0.59–78.91)
Extrapulmonary Complications	<.001	22.94 (3.59–146.67)
Chronic Airway Disease	0.465	1.40 (0.56–3.49)
Lobar Pneumonia	0.005	6.12 (1.75–21.37)
Bronchopneumonia	0.048	0.22 (0.05–0.99)
Allergic History	0.047	0.33 (0.11–0.98)
Pleural Effusion	0.011	6.43 (1.52–27.10)
Family Allergic History	0.989	0.00 (0.00—Inf)
Oxygen Therapy	<.001	7.73 (2.97–20.11)
Corticosteroid Use	<.001	6.22 (2.43–15.93)
Bronchoscopy	0.001	6.94 (2.16–22.29)
Erythrocyte Sedimentation Rate [M(Q1, Q3), mm/h]	0.428	1.02 (0.97–1.07)
Creatine Kinase [M(Q1, Q3), U/L]	0.703	1.00 (0.99–1.00)
CK-MB[M(Q_1,_Q_3_), U/L]	0.039	0.95 (0.91–0.99)
LDH[M(Q_1,_Q_3_), U/L]	0.809	1.00 (1.00–1.00)
Urea Nitrogen [mmol/L]	0.229	0.76 (0.48–1.19)
Creatinine [M(Q_1,_Q_3_), μmol/L]	0.179	1.02 (0.99–1.05)
ALT[M(Q_1,_Q_3_), U/L]	0.486	1.01 (0.99–1.02)
AST[M(Q_1,_Q_3_), U/L]	0.760	1.00 (0.99–1.02)
Albumin [g/L]	0.382	0.93 (0.80–1.09)
D-Dimer [M(Q_1,_Q_3_), mg/L FEU]	0.848	1.07 (0.53–2.17)
CD16+CD56[M(Q_1,_Q_3_),%]	0.105	1.06 (0.99–1.13)
CD19[M(Q_1,_Q_3_),%]	0.720	1.01 (0.96–1.06)
CD3[M(Q_1,_Q_3_),%]	0.226	0.97 (0.93–1.02)
CD3+CD4[μmol/L,%]	0.462	0.97 (0.91–1.04)
CD4[μmol/L,%]	0.635	0.97 (0.85–1.10)
CD8[M(Q_1,_Q_3_),%]	0.501	1.05 (0.91–1.22)
CD4/CD8[M(Q_1,_Q_3_),%]	0.589	0.80 (0.35–1.80)
Serum Amyloid A [M(Q_1,_Q_3_),mg/L]	0.902	1.00 (0.99–1.01)
Procalcitonin [M(Q_1,_Q_3_),ng/ml]	0.858	1.03 (0.78–1.35)
IFN-γ[M(Q_1,_Q_3_),pg/ml]	0.133	1.01 (1.00–1.03)
IL-10[M(Q_1,_Q_3_),pg/ml]	0.239	1.02 (0.98–1.06)
IL-2[M(Q_1,_Q_3_),pg/ml]	0.472	1.10 (0.84–1.45)
IL-4[M(Q_1,_Q_3_),pg/ml]	0.576	1.09 (0.80–1.49)
IL-6[M(Q_1,_Q_3_),pg/ml]	0.093	1.00 (1.00–1.01)
TNF-α[M(Q_1,_Q_3_),pg/ml]	0.058	1.09 (1.00–1.19)
25-Hydroxy Vitamin D [M(Q_1,_Q_3_),ng/ml]	0.277	1.02 (0.99–1.05)
Serum Ferritin [M(Q_1,_Q_3_),ng/ml]]	<.001	1.01 (1.01–1.02)
IgE[M(Q_1,_Q_3_),g/L]	0.036	1.01 (1.01–1.01)
IgA[M(Q_1,_Q_3_),g/L]	0.837	1.07 (0.57–2.02)
IgG[M(Q_1,_Q_3_),g/L]	0.812	1.02 (0.85–1.23)
IgM[g/L]	0.190	0.52 (0.20–1.37)
Leukocyte Count [M(Q_1,_Q_3_),×10^9^/L]	0.548	1.03 (0.93–1.15)
Neutrophil Count [M(Q_1,_Q_3_),×10^9^/L]	0.037	1.03 (1.01–1.07)
Lymphocyte Count [M(Q_1,_Q_3_),×10^9^/L]	0.115	0.97 (0.94–1.01)
Red Blood Cell Count [M(Q_1,_Q_3_),×10^12^/L]	0.927	0.96 (0.38–2.42)
Hemoglobin Concentration [M(Q_1,_Q_3_),g/L]	0.483	0.98 (0.94–1.03)
Platelet Count [M(Q_1,_Q_3_),×10^9^/L]	0.302	1.00 (0.99–1.00)
C-Reactive Protein [M(Q_1,_Q_3_),mg/L]	0.361	1.01 (0.99–1.03)
Fever Duration	0.173	1.13 (0.95–1.35)
Allergen Types	0.589	0.91 (0.65–1.28)
Hospital Stay	0.001	1.46 (1.17–1.84)

#### 3.5.2 ROC curve analysis and cutoff values

**Figure 4:**
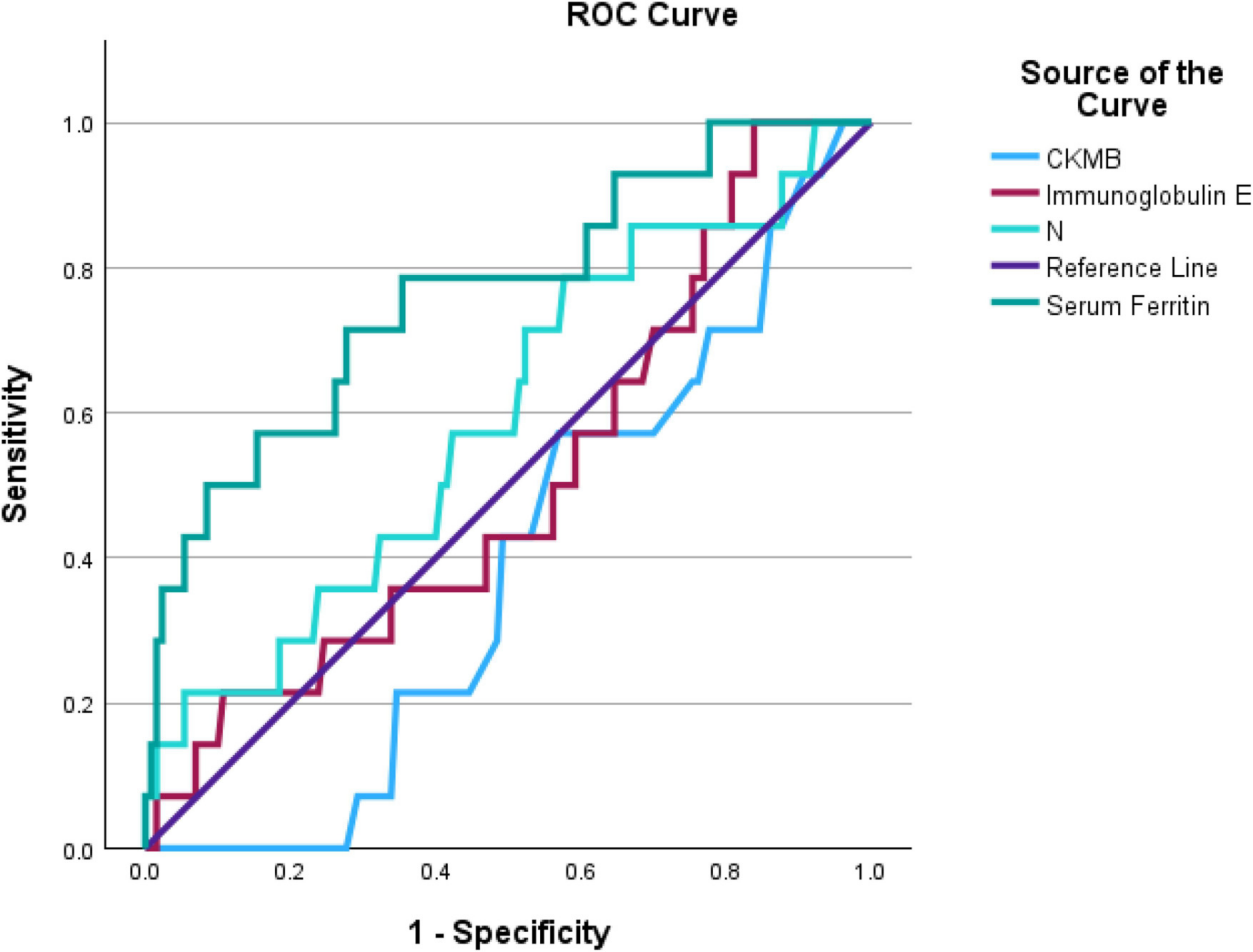
ROC curve for severe Mycoplasma pneumoniae pneumonia in children with atopic constitution [the ROC curve was used to assess the predictive performance of CK-MB, neutrophil percentage (*N*), immunoglobulin E (IgE), and serum ferritin for severe pneumonia in children with atopic constitution].

Quantitative indicators with *P* < 0.05 in the univariate analysis were included in the Receiver Operating Characteristic (ROC) curve analysis to evaluate their predictive value (Figure 4, Table 5). The results showed that serum ferritin had the highest area under the curve (AUC = 0.75, 95% CI: 0.60–0.89), followed by CK-MB (AUC = 0.64) and neutrophil percentage (AUC = 0.63). Based on the Youden index, the optimal cutoff values for serum ferritin, IgE, CK-MB, and neutrophil percentage were 107.61 ng/mL, 1,060 IU/mL, 36.5 U/L, and 59.15%, respectively. When the cutoff value for serum ferritin was set at 107.61 ng/mL, the sensitivity for predicting severe MPP was 73%, and the specificity was 67%.

**Table 5:** Laboratory quantitative indicators for predicting severe MPP in children with atopic constitution.

Item	AUC (95%CI)	Accuracy (95%CI)	Sensitivity (95%CI)	Specificity (95%CI)	PPV (95%CI)	NPV (95%CI)	Cut off
CK-MB[M(Q_1,_Q_3_), U/L]	0.64 (0.53–0.74)	0.61 (0.55–0.67)	0.65 (0.60–0.71)	0.05 (<0.001–0.15)	0.90 (0.86–0.94)	0.01 (<0.001–0.03)	36.5
Serum Ferritin [M(Q_1,_Q_3_),ng/ml]]	0.75 (0.60–0.89)	0.72 (0.65–0.79)	0.73 (0.66–0.80)	0.67 (0.43–0.91)	0.96 (0.92–0.99)	0.20 (0.09–0.31)	107.61
IgE[M(Q_1,_Q_3_),g/L]	0.57 (0.44–0.71)	0.85 (0.80–0.89)	0.89 (0.85–0.93)	0.30 (0.10–0.50)	0.94 (0.91–0.97)	0.18 (0.05–0.31)	1,060
Neutrophil Count [M(Q_1,_Q_3_),×10^9^/L]	0.63 (0.51–0.76)	0.56 (0.50–0.62)	0.55 (0.49–0.61)	0.68 (0.48–0.89)	0.96 (0.92–0.99)	0.11 (0.05–0.16)	59.15

#### 3.5.3 Multivariable logistic regression analysis

Based on the univariate and ROC analyses, all factors with statistical significance were included in a multivariable logistic regression model to identify independent risk factors. The results showed that oxygen therapy (OR = 57.49, 95% CI: 1.69–1,952.29, *P* = 0.024), a history of corticosteroid use (OR = 9.28, 95% CI: 1.19–72.15, *P* = 0.033), serum ferritin > 107.61 ng/mL (OR = 1.01, 95% CI: 1.01–1.02, *P* < 0.05), and IgE > 1,060 IU/mL (OR = 3.16, 95% CI: 1.10–9.07, *P* = 0.032) were independent risk factors for severe MPP in children with atopic constitution (Table 6).

**Table 6:** Multivariable analysis of risk factors for severe Mycoplasma pneumonia in children with atopic constitution.

Factor	Regression coefficient (*β*)	Standard error	Wald value	*P* Value	OR (95%CI)
Dyspnea	−0.91	2.19	−0.42	0.676	0.40 (0.01–29.05)
Extrapulmonary Complications	1.28	2.28	0.56	0.573	3.61 (0.04–314.90)
Lobar Pneumonia	0.64	1.72	0.37	0.709	1.90 (0.07–54.73)
Bronchopneumonia	−18.92	2,229.78	−0.01	0.993	0.00 (0.00—Inf)
Allergic History	0.69	1.66	0.42	0.677	1.99 (0.08–51.60)
Pleural Effusion	1.49	1.39	1.07	0.284	4.44 (0.29–67.61)
Oxygen Therapy	4.05	1.80	2.25	0.024	57.49 (1.69–1,952.29)
Corticosteroid Use	2.23	1.05	2.13	0.033	9.28 (1.19–72.15)
Bronchoscopy	−0.87	1.48	−0.59	0.558	0.42 (0.02–7.67)
Serum Ferritin > 107.61 ng/mL	0.01	0.00	2.03	<0.001	1.01 (1.01–1.02)
IgE > 1,060 IU/mL	1.15	0.54	2.14	0.032	3.16 (1.10–9.07)
Hospital Stay	0.23	0.26	0.88	0.376	1.26 (0.75–2.11)

## 4 Discussion

This study retrospectively analyzed the impact of atopic constitution on the clinical characteristics and prognostic indicators in children with MPP, aiming to provide scientific evidence for clinical diagnosis and treatment of MPP. The results of this study showed that among children with an atopic constitution and MPP, the allergen sensitization status was notably diverse and complex. Although most children were sensitized to a single allergen, a significant proportion (46.8%) had dual or multiple allergen sensitivities, with the number of children with dual sensitization being equal to the number of children with four or more allergens. Bao et al. (8) found that the burden of Mycoplasma pneumoniae (MP) infection in children is positively correlated with a history of allergic diseases, positive results in inhalant allergen tests, and total serum IgE levels. Therefore, this high atopic burden following MPP infection suggests that the immune systems of children have been in a prolonged Th2-dominant sensitized state (9), making it more likely to trigger intense airway inflammatory responses and airway hyperreactivity symptoms. Regarding inhalant allergens, this study found that dust mites (both dust mites and house dust mites) were the most commonly detected allergens, accounting for 34.6% and 31.8% of the samples, respectively. This result aligns with the geographical characteristics of central China, where the climate is humid and promotes dust mite growth. Among food allergens, eggs (36.19%) and milk (35.82%) were the most common allergens. Although food allergies mainly involve gastrointestinal or skin symptoms, the “gut-lung axis” theory in recent years suggests that the interaction between the gut microbiota and the immune system may influence respiratory health (10, 11). Considering the overall distribution of allergens, it is of great importance to identify the specific sensitization types in children with an atopic constitution who are clinically diagnosed with MPP. Clinicians should be particularly vigilant about the risk of wheezing and airway hyperreactivity in children with a high sensitivity to dust mites. In addition to treating MPP, attention should be given to environmental control and allergy treatment interventions. The pathogenesis of MPP primarily involves cellular immunity and cytokine responses, and the host's genetic background can also affect the severity of MPP (12). In other words, atopy may be a risk factor for the severity of Mycoplasma pneumoniae pneumonia and extrapulmonary manifestations in affected children (5, 13). A cohort study involving 7,955 patients found that atopic sensitization and a history of asthma may be risk factors for refractory Mycoplasma pneumoniae infection. The incidence of both early-onset and late-onset asthma is closely associated with Mycoplasma pneumoniae infection, even in non-atopic patients (14). Additionally, the high proportion of multiple sensitizations and food allergies in this study suggests that, beyond the acute management of MPP, long-term allergen avoidance and specific immunotherapy may be important strategies to improve the long-term respiratory health of these children and reduce the sequelae of MPP infection, such as secondary asthma.

The results showed that children in the atopic constitution group exhibited significant differences in clinical symptoms, with the incidence of wheezing, stridor, and dyspnea being significantly higher than that in the non-atopic constitution group (*P* < 0.010 for all). This is consistent with research by domestic scholars (13). This finding indicates that children with an atopic constitution are more likely to develop clinical manifestations of airway hyperreactivity after Mycoplasma pneumoniae infection. As a meta-analysis has stated, patients with a family history of allergies have a greater risk of developing asthma symptoms after Mycoplasma pneumoniae infection (15). The combined effect of Mycoplasma pneumoniae infection and allergies may lead to asthma during childhood. Moreover, a significant proportion of allergic children who are infected with Mycoplasma pneumoniae later develop secondary asthma (16). The mechanisms by which Mycoplasma pneumoniae infection contributes to the development, exacerbation, and worsening of asthma symptoms in children are not yet fully understood. These mechanisms may involve immune responses triggered by stimuli, airway inflammation, IgE-mediated hypersensitivity reactions, and genetic factors in individual patients, such as a family history of atopy, which may help to explain this relationship.

In terms of laboratory tests, this study identified key differences in immune-inflammatory markers between the two groups of children. The total immunoglobulin E (IgE) levels in the atopic constitution group were significantly higher than those in the non-atopic constitution group (*P* < 0.001). Lin Yang (17). Research by Lin Yang and others also supports this finding, as they discovered that higher IgE levels in MPP patients were associated with more severe clinical symptoms and a greater number of complications. A related mechanism suggests that Mycoplasma pneumoniae (MP) induces allergies by producing P1-specific IgE (18). At the same time, the lymphocyte count in the atopic group was significantly lower than that in the non-atopic group (*P* < 0.001), while the tumor necrosis factor-alpha (TNF-α) levels (*P* = 0.048) were significantly higher in the atopic group. In the early stages of MP infection in children, peripheral blood lymphocytes are usually normal or mildly elevated; however, in severe cases or with intense immune inflammatory responses, a decrease in lymphocyte count can occur. Previous studies have reported significantly elevated serum TNF-α levels in children with wheezing, asthma, and acute Mycoplasma pneumoniae infection, which is consistent with the results of this study (15). In addition, the findings of this study are consistent with research by other scholars (5). In contrast, the PCT levels in the atopic constitution group were lower than those in the non-atopic constitution group. PCT is typically used as a specific marker for bacterial infections and their severity, and it significantly increases in cases of typical bacterial infections or sepsis. The lower PCT levels in the atopic constitution group suggest that, although these children exhibited more severe clinical symptoms (such as wheezing and dyspnea), the underlying cause was not primarily due to secondary typical bacterial infections (such as Streptococcus pneumoniae). This may be attributed to Mycoplasma pneumoniae infection rather than a direct reflection of bacterial load.

Further analysis of the comorbidities of chronic airway diseases in children showed that the atopic constitution group had a higher proportion of chronic airway diseases, with a history of wheezing (15.12%) and rhinitis (13.79%) being the most common comorbidities. These results strongly support the idea that, after Mycoplasma pneumoniae pneumonia (MPP) infection, airway hyperreactivity in children with an atopic constitution is activated and amplified, leading to more severe airway obstruction symptoms. Notably, the atopic constitution group included nearly all cases of asthma, obstructive bronchiolitis, and cough-variant asthma in this study. These findings are consistent with existing research that shows that Mycoplasma pneumoniae infection is an important factor in asthma exacerbation, with mechanisms involving airway epithelial damage and enhanced Th2-dominant immune responses. Furthermore, the results suggest that clinicians should pay more attention to airway reactivity in children with an atopic constitution and MPP, who may require more intensive interventions. For example, although the use of oxygen therapy and corticosteroids did not reach statistical significance, the proportions in the atopic group were higher than those in the non-atopic group.

This study used a multivariable logistic regression model to identify the independent risk factors for severe MPP in children with an atopic constitution. The results showed that oxygen therapy (OR = 57.49, *P* = 0.024), a history of corticosteroid use (OR = 9.28, *P* = 0.033), serum ferritin > 107.61 ng/mL (OR = 1.01, *P* < 0.001), and immunoglobulin E (IgE) > 1,060 IU/mL (OR = 1.00, *P* = 0.007) were independent risk factors. Oxygen therapy and a history of corticosteroid use may serve as markers of disease severity and treatment resistance. Literature reports suggest that timely corticosteroid use during the treatment of exacerbated MPP with an overactive immune response is a reasonable treatment option (19). Serum ferritin and immunoglobulin E (IgE), as laboratory markers, have significant predictive value. ROC curve analysis revealed that the area under the curve (AUC) for serum ferritin (AUC = 0.75, 95% CI: 0.60–0.89) had the highest predictive value, with an optimal cutoff value of 107.61 ng/mL. This finding suggests that serum ferritin may play an important role in the progression to severe MPP in children with an atopic constitution and could serve as a reliable biomarker for the early prediction of severe MPP. Other studies have also indicated that hospitalized children diagnosed with Mycoplasma pneumoniae-related extrapulmonary diseases have significantly higher serum IgE levels than those with only respiratory diseases (20).

Although this study highlights the significant impact of atopic constitution on the clinical features and severity risk of MPP in children, it has some limitations that should be considered. This was a single-center retrospective study with data sourced from a single medical institution and a relatively limited sample size. Additionally, the study primarily focused on acute clinical manifestations and laboratory markers during hospitalization, lacking long-term follow-up data after discharge. Therefore, it is not possible to further assess the long-term effects of atopic constitution on pulmonary function recovery, the incidence of secondary asthma, and airway remodeling in children with MPP. Based on these limitations, future research directions could focus on the following aspects: first, conducting multicenter, large-sample prospective cohort studies to verify the stability and universality of the independent risk factors identified in this study (such as serum ferritin and IgE levels) for predicting severe MPP and to establish a more accurate model for predicting severe disease risk. Second, in-depth mechanistic studies could explore how the coexistence of atopy and/or respiratory allergies may predispose individuals to extrapulmonary complications associated with acute Mycoplasma pneumoniae infection (21).

## 5 Conclusion

This study revealed the unique characteristics of children with atopic constitution and MPP in terms of clinical symptoms, comorbidities, and immune-inflammatory markers. This study indicates that an atopic constitution is closely associated with increased clinical manifestations of airway hyperreactivity in children with MPP. Clinically, oxygen requirements, history of corticosteroid use, serum ferritin, and IgE levels should be considered key indicators for assessing the risk of severe MPP in children with an atopic constitution. In particular, the cutoff values for serum ferritin and IgE can provide quantitative guidance for clinicians, helping to identify high-risk children early and enabling active monitoring and intervention, thereby improving treatment outcomes and prognosis of these children. These findings highlight the role of the underlying atopic immune status in the progression to severe MPP, suggesting that clinicians should focus on preventing disease progression and minimizing the development and evolution of post-infection complications in children with an atopic constitution.

## Data Availability

The original contributions presented in the study are included in the article/Supplementary Material, further inquiries can be directed to the corresponding author/s.
