# Supplementary figures and images for: Clinical characteristics of Mycoplasma pneumoniae pneumonia in children with atopic constitution and risk factors for disease severity: a retrospective comparative study
Source: Front Pediatr. 2026 Apr 10;14:1760932. doi: 10.3389/fped.2026.1760932 (PMC13106515; doi:10.3389/fped.2026.1760932)

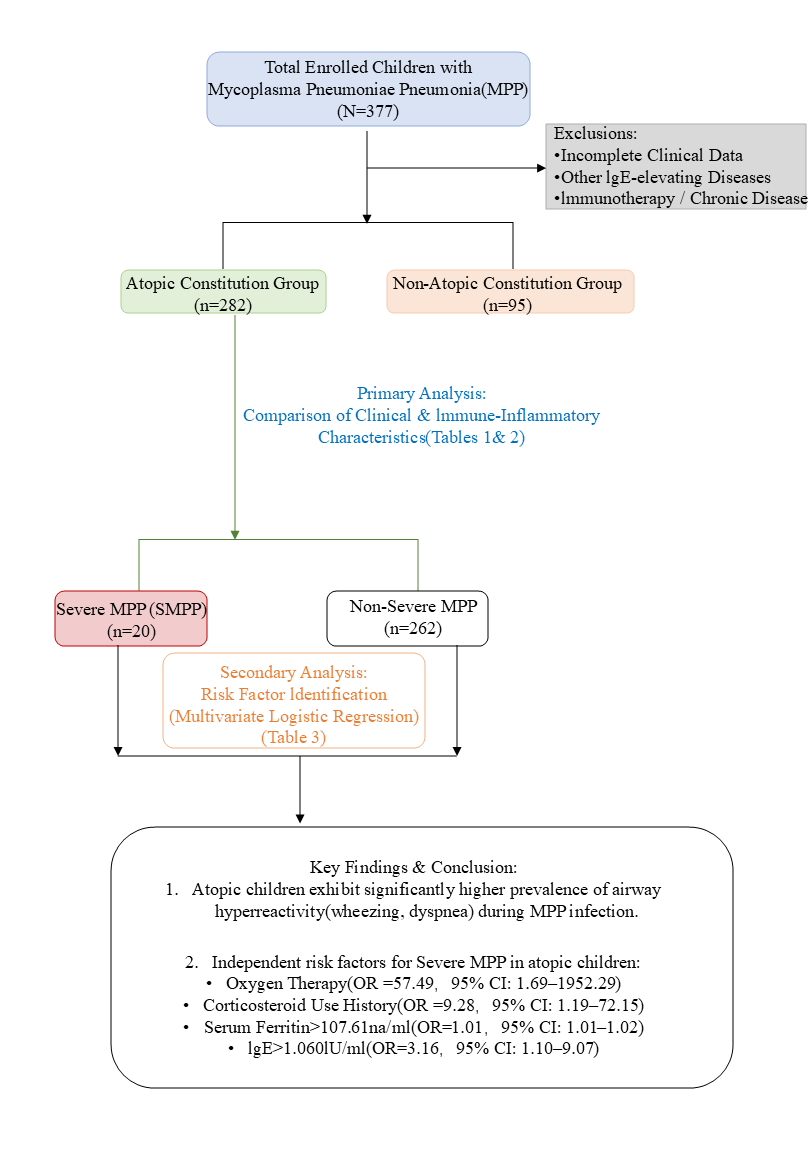

Supplement: Supplementary file 1 [file Image1.tiff]
